# Hydrogen Gas Alleviates Chronic Intermittent Hypoxia-Induced Renal Injury through Reducing Iron Overload

**DOI:** 10.3390/molecules24061184

**Published:** 2019-03-26

**Authors:** Peng Guan, Zhi-Min Sun, Li-Fei Luo, Ya-Shuo Zhao, Sheng-Chang Yang, Fu-Yang Yu, Na Wang, En-Sheng Ji

**Affiliations:** 1 Department of Physiology, Hebei University of Chinese Medicine, Shijiazhuang 050200, Hebei, China; guanpeng@hebcm.edu.cn (P.G.); sunzhimin@hebcm.edu.cn (Z.-M.S.); luolifei@hebcm.edu.cn (L.-F.L.); yangshengchang@hebcm.edu.cn (S.-C.Y.); yufuyang@hebcm.edu.cn (F.-Y.Y.); wangna@hebcm.edu.cn (N.W.); 2 Scientific Research Center, Hebei University of Chinese Medicine, Shijiazhuang 050200, Hebei, China; zhaoyashuo@hebcm.edu.cn

**Keywords:** hydrogen, chronic intermittent hypoxia, kidney, iron

## Abstract

Iron-induced oxidative stress has been found to be a central player in the pathogenesis of kidney injury. Recent studies have indicated H_2_ can be used as a novel antioxidant to protect cells. The present study was designed to investigate the protective effects of H_2_ against chronic intermittent hypoxia (CIH)-induced renal injury and its correlation mechanism involved in iron metabolism. We found that CIH-induced renal iron overloaded along with increased apoptosis and oxidative stress. Iron accumulates mainly occurred in the proximal tubule epithelial cells of rats as showed by Perl’s stain. Moreover, we found that CIH could promote renal transferrin receptor and divalent metal transporter-1 expression, inhibit ceruloplasmin expression. Renal injury, apoptosis and oxidative stress induced by CIH were strikingly attenuated in H_2_ treated rats. In conclusion, hydrogen may attenuate CIH-induced renal injury at least partially via inhibiting renal iron overload.

## 1. Introduction

Obstructive sleep apnea (OSA) affects more than 100 million adults all over the world [1]. Now, OSA has been recognized as one of the important causes or factors of worsening for various renal injury [2], cardiovascular diseases [3], cerebrovascular damage [4], asthma [5], and pulmonary edema [6]. Several theories have been developed to explain the excess risks among individuals with OSA, including oxidative stress, hypoxemia, inflammation, and hypertension [2].

In the literature, the relationship between OSA and chronic kidney disease (CKD) seems bidirectional. OSA appears to have a high occurrence in CKD patients who require dialysis, the ratio has shot up to one-third of their total numbers [7]. Especially, occurrence of OSA in people with end-stage renal disease (ESRD) reaches about 50–70%, which suggests that OSA is common in these patients [2]. A retrospective cohort study showed a similar effect that OSA was 1.94 times higher in CKD patients, and this had risen to 2.2 times in ESRD patients [8]. 

Hydrogen gas (H_2_) is a biomedical agent with antioxidative, anti-inflammatory and anti-allergic properties [9]. H_2_ has favorable cellular bio-availability as it can rapidly diffuse into cells to play a beneficial role, given the physical properties. More importantly, H_2_ has no inhibitory effect on metabolic redox function and reactive oxygen species (ROS) that acts a signaling molecule. H_2_ can also be anti-apoptotic and protective by inducing hormesis or pre-condition like cellular states. [10].

Iron is a required element in the body that acts as a component of many enzymes to exert its physiological actions [11]. However, excessive iron could accelerate the production of damaging ROS, leading to lipid peroxidation and DNA mutagenesis [12]. The delivery of transferrin bound iron to cells is through transferrin–transferrin receptor interaction on the cell surface. Following internalization of the complex by clathrin-mediated endocytosis, iron traverses from the plasma membrane to enter the cytosol [13]. When transferrin saturation is exceeded, non-transferrin bound iron uptake will take place through divalent metal transporter 1 (DMT1) and ferroportin (FPN). The DMT1 distribution was observed in mice by specific immunoreactivity in proximal tubules, suggesting that DMT-1 may function on apical iron entry [14]. As the sole cellular iron efflux channel identified to date in vertebrates, FPN extrudes iron from the cells [15]. FPN expressed in proximal tubule cells of rats and may provide a means of iron exit [16]. Iron overload may develop kidney impairment and can be an early and sensitive indicator in CIH-induced kidney disease. Wang et al. assessed the probability of iron deposition in patients undergoing renal biopsy, they found thirty-four of them showed definite iron staining [17]. Moreover, it has been shown that iron is often sequestered in cells in CKD patients [18]. Wareing et al. found that serum iron is available for glomeruli to filter, even the majority of the filtered iron is followed by tubular reabsorption [19]. 

However, in spite of these particular studies, it is not known whether H_2_ can reduce the renal injury caused by CIH. Furthermore, not much has reported about the alterations of renal iron metabolism under CIH. Thus, we do not know whether H_2_ protect kidney against CIH-induced injury via regulating iron metabolism. Therefore, this study was performed to evaluate the effect of H_2_ treatment against the deleterious renal consequences of CIH and the possible pathway it involves.

## 2. Results

### 2.1. H_2_ Alleviates the Injury of Kidneys in Rats

Compared with the control group, in the CIH group, glomerular and renal cysts and renal tubular epithelial cells showed clearly swollen as lumen was narrow or nearly closed. However, these alterations were notably alleviated by H_2_. There was no obvious renal morphological change in the CON + H_2_ group.

**Figure 1:**
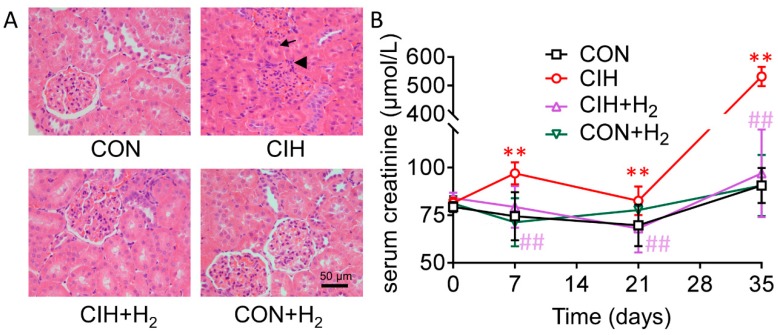
Effect of H_2_ on the structure and function of kidney in rats exposed to CIH (chronic intermittent hypoxia) (**A**). Representative hematoxylin and eosin stained images. CIH-induced remarkable renal structure damage, such as glomerular and renal cysts and renal tubular epithelial cell swelling. These alterations were notably alleviated by H_2_. Arrow, swollen proximal tubule. Arrow head, glomerulus. (**B**). Rats treated with CIH showed increase of serum creatinine levels on 7, 21 and 35 days. A significant decrease occurred in CIH + H_2_ group compared to corresponding CIH group. There was no significant change use H_2_ alone. ** *p* < 0.01 vs. normoxia group; ## *p* < 0.01 vs. CIH group.

Serum creatinine level is an important marker of kidney injury as it inversely correlates with the glomerular filtration rate. No statistically significant differences were found among the four groups before initiation of the CIH and/or H_2_. Serum creatinine levels were significantly higher in CIH rats on 7th, 14th, and 35th day as compared with the corresponding control group (each *p* < 0.01). Notably, increased creatinine level was normalized by H_2_ (each *p* < 0.01). H_2_, when treated alone, had nearly no effect on creatinine levels (Figure 1B).

### 2.2. H_2_ Inhibits CIH-Induced Renal Apoptosis

**Figure 2:**
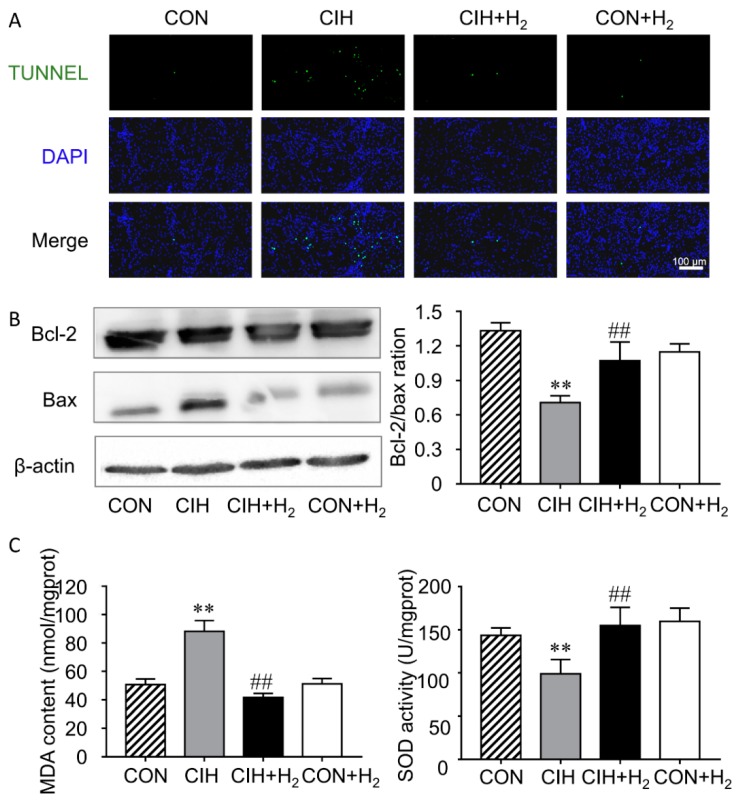
Effect of H_2_ on CIH-induced renal cell apoptosis and oxidative stress. (**A**). Representative images of cell apoptosis. (**B**). Western blot showing that H_2_ inhibited the decrease of Bcl-2/bax ratio. (**C**). H_2_ administration suppressed renal MDA (malondialdehyde) content and increased SOD (superoxide dismutase) activity. ** *p* < 0.01 vs. normoxia group; ## *p* < 0.01 vs. CIH group.

Compared with the control group, the cell apoptosis rate was increased in the CIH group. However, the renal cell apoptosis was significantly suppressed with H_2_ inhalation but H_2_ alone had no effect (Figure 2A).

A major checkpoint in apoptosis is the expression of anti-apoptotic proteins to pro-apoptotic proteins. Here we used Western blotting to examine the ratio of Bcl-2 and Bax. CIH-induced decreased Bcl-2/Bax ratios compared to control (*p* < 0.01). Bcl-2/Bax ratios increased significantly after H_2_ inhalation in CIH + H_2_ group (*p* < 0.01). Treatment of H_2_ alone showed no apparent change in the Bcl-2/Bax ratios (Figure 2B).

### 2.3. H_2_ Reduces CIH-Induced Renal Oxidative Stress

To examine whether the increased level of oxidative stress was responsible for CIH-induced cell apoptosis, MDA level as well as SOD (superoxide dismutase) activity were determined. The data showed that, the renal MDA level was significantly increased after suffering from CIH, whereas that of SOD activity decreased significantly. H_2_ decreased MDA levels and elevated the activity of SOD (Figure 2C).

### 2.4. H_2_ Alleviates CIH-Induced Renal Iron Overload

To determine whether the iron-dependent lipid peroxidation plays a role in increased oxidative stress, the distribution and content of iron was detected in rat kidneys. 

**Figure 3:**
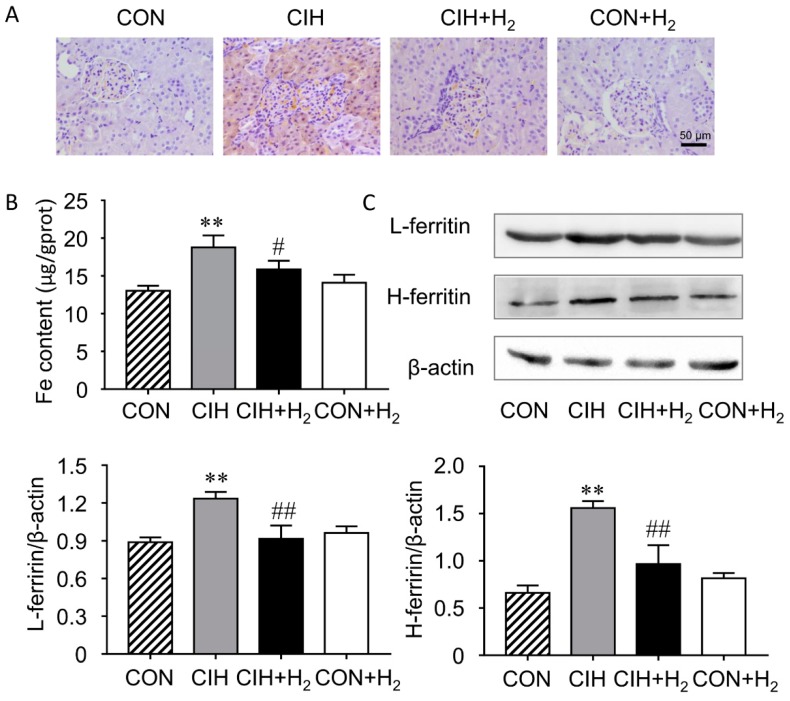
Effect of H_2_ on CIH-induced renal iron overload. (**A**). Representative images of DAB (3,3′-diaminobenzidine) enhanced Perl’s Prussian blue staining. Iron is shown to deposit in proximal tubule epithelial cells. (**B**). The contents of renal non-heme iron were determined by colorimetry. (**C**). Western blot showing that H_2_ inhibited the increase of both FtL and FtH expression. ** *p* < 0.01 vs. normoxia group; # *p* < 0.05, ## *p* < 0.01 vs. CIH group.

There is a handful of scattered iron in the control group. Obviously, iron deposition in the proximal tubules of the kidneys was discovered in the CIH group. After H_2_ administration, the specific iron deposition was inhibited in the CIH + H_2_ group (Figure 3A).

The content of iron in the kidney was confirmed by tissue iron assay kit from the Nanjing Jiancheng (Figure 3B). The iron content in the kidney increased significantly in the CIH group compared to control (*p* < 0.01). In the CIH + H_2_ group, the iron deposition was inhibited by H_2_ (*p* < 0.05).

Ferritin is a protein that stores iron. The results showed that the protein expression of FtL and FtH was increased significantly in the CIH group by 39% and 134% respectively when compared with control (each *p* < 0.01), indicating a state of iron overload. Decreased FtL and FtH protein expression level to 73% and 62% respectively was found in the CIH + H_2_ group (each *p* < 0.01), whereas no obvious changes of FtL and FtH were seen in rats treated with H_2_ alone (Figure 3C).

### 2.5. H_2_ Suppresses CIH-Induced Increase Expression of Renal TfR and DMT1 and Decrease of CP

**Figure 4:**
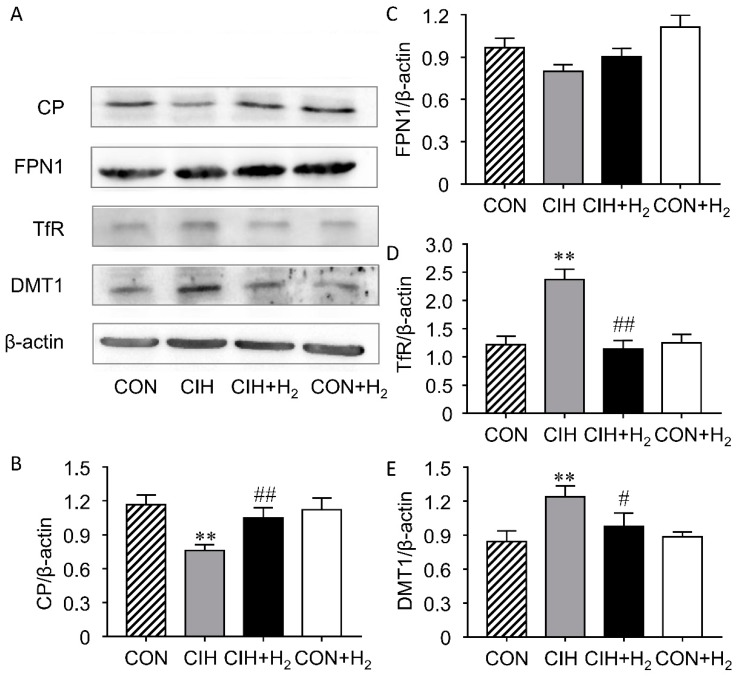
Effect of H_2_ on renal iron transporting related proteins expression. H_2_ administration alleviates mesenteric arterial dysfunction in CIH rats. (**A**). Representative Western blot photographs. (**B**). Renal CP (ceruloplasmin) expression. (**C**). Renal FPN (ferroporrtin) expression. (**D**). Renal TfR expression. (**E**). Renal DMT1 (divalent metal transporter 1) expression. ** *p* < 0.01 vs. normoxia group; # *p* < 0.05, ## *p* < 0.01 vs. CIH group.

To evaluate the change of iron release and uptake of iron from kidney, we determined the levels of CP (ceruloplasmin), FPN, TfR, and DMT1 using Western blots (Figure 4A). CP expression was significantly reduced to 65% after a CIH exposure for 5 weeks in rats, while H_2_ significantly enhanced the protein expression of CP by 37% (Figure 4B). However, our measure of renal FPN expression showed no significant differences among the four groups (Figure 4C). Was the increased iron reabsorption from tubular fluid responsible for CIH-induced iron accumulation? Here, the TfR level doubled in the kidney from the CIH groups compared with control (*p* < 0.01), suggesting an elevation in transferrin dependent iron uptake (Figure 4D). Moreover, CIH-induced DMT1 up-regulation by 46% was also seen in the kidneys (Figure 4E). The results indicated that CIH-induced higher non-transferrin-bound iron uptake than that in the control rats. In contrast, H_2_ inhalation reduced both TfR and DMT1 protein expression to 47% and 79% respectively in CIH + H_2_ group. Additionally, it was noted that the H_2_ treated alone group showed no notable difference in the expression of TfR and DMT1 compared to control.

### 2.6. H_2_ Normalizes Renal HIF-1α and HO-1 Expression Levels

**Figure 5:**
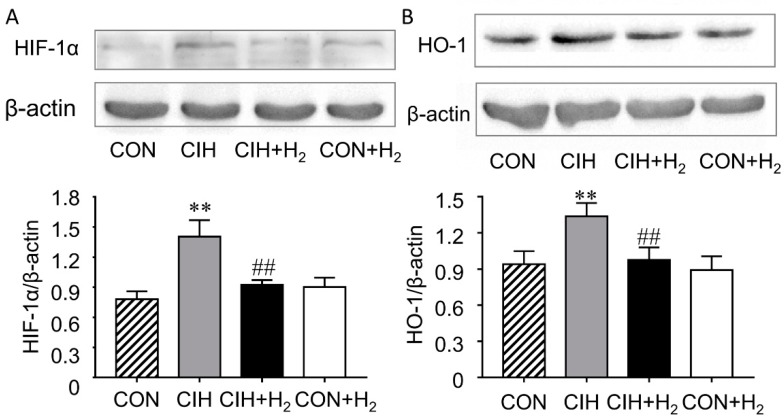
Effect of H_2_ on renal HIF-1α (hypoxia-inducible factor-1α) and HO-1 expression. (**A**). H_2_ inhibited the increase of HIF-1α expression. (**B**). H_2_ suppressed the increase of HO-1 expression. ** *p* < 0.01 vs. normoxia group; ## *p* < 0.01 vs. CIH group.

Hypoxia-inducible factor (HIF-1) can upregulate several genes with hypoxia responsive elements (HREs) in the promotors by induce transcription. The results showed that protein expression of HIF-1α was significantly elevated by 79% in the CIH group while H_2_ effectively eliminated the elevation to 65%. Besides, the H_2_ alone group showed no notable difference when compared to control (Figure 5A).

The change of HO-1was in line with the protein levels of HIF-1α (Figure 5B). CIH rats exhibited elevated HO-1 expression by 43% than that of the control rats. Importantly, the increase of CIH-induced HO-1 protein expression was reversed by administration of H_2_ in the CIH + H_2_ group (to 73%). Besides, H_2_ treated alone group showed no notable difference in the expression of HO-1 compared to control.

## 3. Discussion

In this study, we examined whether H_2_ can protect the kidney against CIH-induced injury. The results confirmed that CIH could damage the structure and function of the kidney, a link already demonstrated by primary studies [20,21]. Our current study shows that excessive iron may participate in the CIH-induced kidney cell injury. Furthermore, we discovered that H_2_ inhalation prevented progression of CIH-induced kidney injury by inhibiting iron accumulation in proximal tubule cells.

Histological examination confirmed that CIH induced renal injury. Intermittent hypoxia, characterized by cyclical changes in hypoxemia with reoxygenation, contributes to generation of too much reactive oxygen species (ROS) [22]. In view of this, we speculated that antioxidants might lower the risk of CIH-induced renal injury. However, many antioxidant supplements increased mortality instead of preventing cancer, myocardial infarction and atherosclerosis [23]. It is reported that H_2_ can selectively reduce toxic reactive oxygen species [24]. Given that no side effects on the use of hydrogen have been reported until now, we thought that the profiling result could probably be used to alleviate the CIH-induced renal injury more efficiently. Our hypothesis was convinced by our finding that H_2_ significantly improved kidney pathological changes in rats. There was not any differential pathology change in the kidney of H_2_ group, confirmed that H_2_ had no major side effects. Serum creatinine, generally used for estimation of the glomerular filtration rate, is a predictor marker of kidney function. Recently, we and others reported that CIH caused an elevated serum creatinine in CIH groups compared to control [25]. Another important result of the current study was that H_2_ inhibited glomerular filtration rate, suggesting that H_2_ can improve renal function.

Apoptosis is known as a critical pathological process in CIH-induced cell injury, and inhibiting apoptosis could relieve CIH-induced injury in kidney [26], heart [27,28], aortic endotheliocyte [29], hippocampus [30] and so on. H_2_ treatments overwhelmingly reduced apoptosis rate of renal tubule cells in rats exposed to CIH in this study, which was consistent with previous studies. The benefit of H_2_ treatments was found to reduce the kidney injury of burn via inhibiting apoptosis and inflammation induced by lipid peroxidation [31]. Besides, in another study about whether hydrogen could protect against kidney injury following orthotropic liver transplantation, hydrogen-rich saline dramatically alleviated renal injury via its antioxidant capability [32].

Our recent and other studies suggested that H_2_ inhalation could ameliorate CIH-induced oxidative stress in rats [30,33]. To further illustrate the related molecular mechanism for apoptosis in the CIH-induced kidney injury, we examined the oxidative stress level. In the CIH groups, the MDA levels were significantly increased, whereas the SOD activities were reduced significantly. Here, the application of H_2_ increased the activity of SOD, and decreased MDA expression in the kidney. It implied that the increased level of oxidative stress was responsible for CIH-induced cell apoptosis, whereas H_2_ markedly reduces the danger of kidney damage by buffering the oxidative stress. Previous studies suggested that chronically renal iron exposure would cause tubular injury and increase in glomerular permeability by interfering the redox balance [34]. The change of oxidative stress in this study corresponding with the iron change in the kidney excited our interest.

There is a labile iron pool (LIP) that seems accessible for use in the cell and for transport to the bloodstream via ferroportin [35]. The increase iron pool would give rise to oxidative stress and protectants like H_2_ likely induce hormesis-like effects. We therefore examined whether increased iron was present in the kidneys of CIH-exposed rats. Elevated levels of iron in kidney homogenates were confirmed in CIH rats by colorimetry. Our results indicated H_2_ restrained the growth of iron level in the kidney of rats when exposed to CIH. Note that iron accumulation is limited to renal proximal tubules as the Perl’s staining shows, meaning that proximal tubules might account for most of the reabsorption of iron from tubular fluid. Previous studies showed that iron in the tubular lumen was nearly reabsorbed again by renal tubular cells, as there were undetectable iron in the healthy urine [36]. Also, with regards to mitochondrial content and energy consumption, proximal tubules were mainly attacking targets of renal damage [37]. Based on these findings, we believe that CIH-induced iron overload in proximal tubules may be involved in the onset of kidney damage. Ferritin consists of two subunits (H and L) and is typically induced by increased iron content. In this study, the expressions of both H-ferritin and L-ferritin is in accordance with iron contents in kidneys.

It is widely believed that the kidney is a key organ involved in homeostasis of iron concentration. [38]. Ferroportin is one of the rate-limiting proteins as it is the way to get rid of iron with the aid of the multicopper ferroxidase ceruloplasmin [35]. So, the changes in the ferroportin–ceruloplasmin system probably could produce such large changes in iron metabolism. Overexpression of transferrin receptor and DMT1 could explain an iron overload of renal epithelial cells. Alternatively, the transferrin receptor and DMT1 mRNA translation should be activated via iron regulatory proteins under iron deficient [39]. That was contradictory to the existing fact that iron overloaded. Indeed, renal iron metabolism is a tightly regulated process as demonstrated by recent studies. Zhang et al. found that the TfR expressed in the proximal tubule epithelial cells [40], may function in Tf-bound iron uptake from glomerular filtrate. As the major protein for iron absorption from the duodenum, DMT1 expresses in the proximal tubules mainly [41], distal tubules and collecting ducts [42]. However, the expression of DMT-1 is located at lysosomal membranes and the late endosomal of proximal tubule cells, suggesting the main function of DMT1 in the proximal tubule is for facilitating Tf-bound iron uptaking [43]. In another experiment, DMT-1 is found also expressed in distal tubules, where it can take part in iron resorb when too much iron remains [40]. Our results found that TfR and DMT1 protein expression was notably increased in the CIH group, indicating that elevated transferritin-dependent iron uptake was involved in the renal iron overload induced by the CIH treatment. H_2_ effectively reduced iron uptake as through decreasing the protein expression of both TfR and DMT1. The observation is in line with the current knowledge that TfR [44] and DMT1 [45] are hypoxia-inducible genes with the putative hypoxia responsive element (HRE) sequence. It is generally accepted that FPN acts with the help of the ferroxidase to mediate iron export, loss of its activity will result in cellular iron overload [46]. However, FPN was reported to facilitate iron reabsorption under basal conditions express as it expressed in the proximal tubules. Additionally, elevated FtH expression could change the location and expression in the tubule [47]. In this study, we demonstrated that FPN expression had no statistically significant increase with CIH administration. As the transcription initiation site of FPN contained putative HREs [48], the increased HIF-1α induced by CIH may positively regulate the amount of FPN. Hepcidin may negatively regulate the expression of FPN as increased hepcidin levels were discovered in patients with OSA [49]. Excessive hepcidin interference with the release of dietary iron and recycled iron in CKD patients by downregulating FPN expression [50]. Thus, the balance of inhibitors and activators tunes the expression of FPN. Redistribution of FPN from tubular apical membrane to the basolateral membrane induced by elevated FtH expression in CIH group should be more meaningful for iron release in renal epithelial cells. Furthermore, the enhanced iron release ability is not large enough to offset the increased iron uptake in CIH rats and showed renal iron overload. Ferroxidase activity is needed to maintenance iron efflux function of ferroportin in some cell types [51]. The protein expression of CP was dramatically decreased in the CIH group than control, making it likely that reabsorbed iron retained in the renal tubular epithelial cells. H_2_ significantly prevents the decreased level of renal CP both in the CIH + H_2_ group and the CON + H_2_ group. The promotion of renal CP level by H_2_ may be due to the antioxidant capabilities as ROS is reported to promote CP mRNA decay by disturbing RNA-protein complex formation [52].

HIF-1 can activate its target genes by transcription [53]. In this study, higher HIF-1α levels induced by CIH may be associated with the increased TfR and DMT1 whose transcription initiation site contained putative HREs. We concluded that H_2_ may decrease TfR and DMT1 expression in CIH rats via regulating the expression of HIF-1α.

HO-1 is reported to be increased by HIF-1α and chronic hypoxia [54]. Previous studies have demonstrated that HO-1 can breakdown heme and increases the availability of its degradation products iron [55]. Thus, the increased renal expression of HO-1 in CIH rats is likely to contribute to iron overload via a cascade of oxidative reactions resulting in the loss of heme. Additionally, administrating H_2_ during CIH may inhibit excessiveness of iron extract from heme.

**Figure 6:**
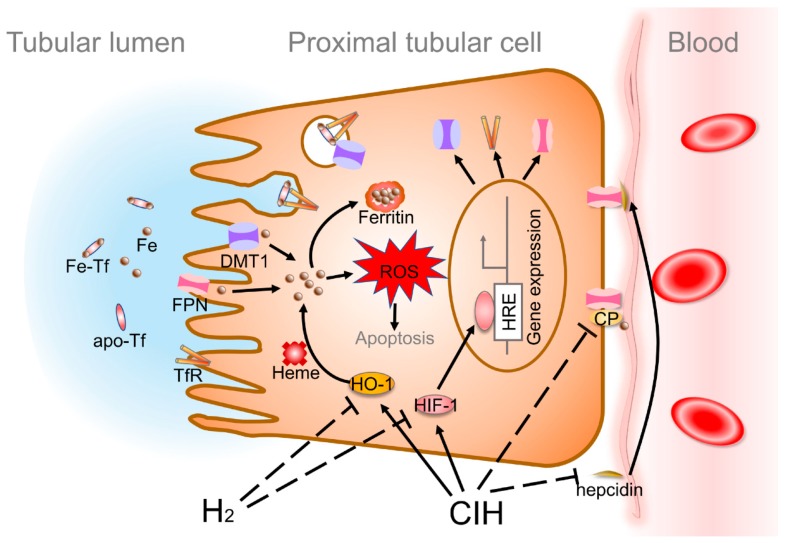
Proposed signaling pathways involved in the protective effect of H_2_ in CIH-induced renal injury. The renal injury, apoptosis and oxidative stress induced by CIH were strikingly attenuated in H_2_ treated rats. Mechanistically, hydrogen gas attenuated CIH-induced elevated expression of HIF-1α, and downregulation of HIF-1α blunted its binding to the hypoxia-response element (HRE) DNA sequence of target proteins such as TfR and DMT1. FPN is an oxygen-responsive gene that affected by HIF-1 and mediated by the iron-regulatory hormone hepcidin. The current renal FPN expression should be a balance of hypoxia and hepcidin. Meanwhile, CIH inhibit CP expression via promoting its mRNA decay. What’s more, H_2_ suppressed CIH induced iron extracted from heme by decreasing HO-1 expression.

In conclusion, the results presented here give a support to a sequence of events whereby iron overload participated in CIH-induced renal injury through oxidative stress. CIH could promote renal TfR and DMT1 expression, as they are oxygen-responsive genes with HREs sequence within their promoter. FPN is an oxygen-responsive gene that is affected by HIF-1 and mediated by the iron-regulatory hormone hepcidin. The current renal FPN expression should be a balance of hypoxia and hepcidin. Moreover, CIH inhibit CP expression via promoting its mRNA decay. What’s more, CIH-induced iron extracted from heme by increasing HO-1 expression. Thus, iron overloaded proximal tubule epithelial cells may suffer from oxidative stress and apoptosis through generating both hydroxyl radicals and higher oxidation states. H_2_ could inhibit iron overload in the renal tubule epithelial cells, and alleviate renal injury, potentially through regulating HIF-1 and HO-1, suggesting iron metabolism might be a therapeutic target in future treatment of OSA (Figure 6).

## 4. Materials and Methods

### 4.1. Animals and Experimental Design

All animal research was approved by the Bioethics Committees of Hebei University of Chinese Medicine. Male Sprague–Dawley rats (weight, 200 ± 10 g) were obtained from the Vital River Laboratory Animal Technology Co., Ltd. (Beijing, China). These rats were maintained on a 12 h light and 12 h dark cycle. Animals were provided free access to water and diet. All animal studies in this study were approved by the Bioethics Committees of Hebei University of Chinese Medicine, the ethic approval number is HEBCM-2018-019.

Twenty four rats were allocated into four groups: Control, CIH, CIH + H_2_, and CON + H_2_ group. Exposure of animals to CIH is similar to that described before [56]. Briefly, unrestrained, freely moving rats were placed in intermittent hypoxic chambers (Oxycycler model A84XOV, BioSpherix, Lacona, NY, USA) with electronically regulated solenoid switches that controlled the O_2_ concentration by a supply of oxygen and nitrogen. The rats in CIH and the CIH + H_2_ group were exposed to 21% to 9% of inspired oxygen (FiO_2_) every 3 min. The hypoxic pattern was repeated for 8 h/day in a total of 35 days. The rats in control and CON + H_2_ group were subjected to identical exposure conditions with room air. The rats in H_2_ treatment groups inhaled H_2_ for 2 h daily after CIH exposure through a ventilator circuit. H_2_ was produced by a hydrogen-Oxygen nebulizer (AMS-H-01, Shanghai Asclepius Meditec Co., Ltd., Shanghai, China).

### 4.2. Histopathological Examinations

Kidney tissues were collected and fixed in 4% paraformaldehyde for 24 h and then followed by embedding in paraffin. Hematoxylin-eosin (HE) staining was carried out on 4 μm thick sections for general cell morphology. The sections were examined by Leica DM2500M microscope (Leica Microsystems, Wetzlar, Germany).

### 4.3. Assay of Serum Creatinine Level

Blood samples were collected by retroorbital bleeding and then let stand at room temperature for 15 min. Following centrifugation at 1500 rpm for 15 min, serum was isolated and was stored at −20 °C until usage. Serum creatinine were detected using commercial kit (Jiancheng Institute of Biotechnology, Nanjing, China) according to the protocol described previously [57].

### 4.4. Cell Apoptosis Assay

Cell apoptosis was determined using terminal dUTP nick end-labeling (TUNEL) assay with an in situ cell death detection kit (Roche Applied Science, Mannheim, Germany). Deparaffinized 4 μm thick sections were treated with proteinase K (20 μg/mL in PBS), and then the sections were incubated with TUNEL-reaction mixture at 37 °C for 1 h. Subsequently, the tissue sections were washed with phosphate-buffered saline followed by staining of the nuclei with DAPI. All sections were examined by Leica DM2500M microscope (Leica Microsystems, Wetzlar, Germany).

### 4.5. Measurements of Malondialdehyde (MDA) and Superoxide Dismutase (SOD) Activity

Oxidative stress was investigated in renal cortex homogenates by commercial kit (Jiancheng Institute of Biotechnology, Nanjing, China) to measure the levels of MDA and SOD activity according to the methods of Ohkawa et al. [58] and Nishikimi et al. [59].

### 4.6. Assay of Renal Iron Level

The commercial kit (Jiancheng Institute of Biotechnology, Nanjing, China) was used to measure renal iron level according to Xie et al. [60]. Briefly, Fe^3+^ was reduced to Fe^2+^ in an acidic buffer solution, after which Ferene S reacted with Fe^2+^ to produce a blue color. The absorbance was recorded using a microplate spectrophotometer (Varioskan LUX, Thermo Scientific, Rockford, IL, USA) at 520 nm.

Iron levels and distribution in the kidneys were determined by Perls’ Prussian blue staining with modifications [61]. Assays were performed as follows: The sections were first treated with 0.3% H_2_O_2_ in methanol for 30 min to block endogenous peroxidase activity. The slides were incubated with a freshly made Perl’s solution (4% potassium ferrocyanide:4% hydrochloric acid = 1:1) at 37 ℃ for 12 h and then followed 3,3′-diaminobenzidine (DAB) stain (Zhongshan Golden Bridge, Beijing, China) for 15 min. Hematoxylin was then used to counterstain the cell nuclei.

Iron overload was also assessed using Western blots by L-ferritin (FtL) and H-ferritin (FtH) expression levels.

### 4.7. Western Blot Analyses

The protein expression values of DMT1, FPN1 and CP were detected using Western blot analysis. Protein was extracted from renal cortex samples by homogenizing with RIPA lysis buffer. The total protein concentration was detected by Bicin-choninic Acid Assay Kit (Beijing Kangwei Century Biotechnology, Beijing, China). Antibodies against FtL, FtH, TfR, HIF-1α, HO-1 and β-actin were supplied by Abcam (Hong Kong, China), while the antibodies against DMT1, FPN1 and CP were supplied by Alpha Diagnostic International (San Antonio, TX, USA). The immunoreactive bands were detected on a Chemiluminescence Analyzer (LAS-3000, Fujifilm, Tokyo, Japan) with enhanced chemiluminescence reagent (Beijing Kangwei Century Biotechnology, Beijing, China).

### 4.8. Statistical Analysis

All quantitative results were presented as mean ± standard deviation (SD). Independent Student’s t-test was used for comparing 2 groups and one-way ANOVA followed by Bonferroni post-test for multiple comparisons using GraphPad Prism v. 6.0 (GraphPad Software, Inc., La Jolla, CA, USA). Statistical significance for all data was *p* < 0.05.

## 5. Conclusions

Increased expression of renal transferrin receptor and divalent metal transporter-1 expression, as well as inhibited ceruloplasmin expression may be possible causes of iron accumulation in the proximal tubule epithelial cells of rats after CIH treated. Inhalation of H_2_ could prevent renal injury induced by CIH; this protection may be associated with inhibition of iron overload in renal epithelial cells, which was involved in the mechanism of oxidative stress-mediated injury.
